# Supplementary material for: Dynamic acidity in defective UiO-66
Source: Chem Sci. 2016 Mar 22;7(7):4706–12. doi: 10.1039/c5sc04953a (PMC6016445; doi:10.1039/c5sc04953a)
Supplement: SC-007-C5SC04953A-s004 [file SC-007-C5SC04953A-s004.pdf]

Electronic Supplementary Material (ESI) for Chemical Science.
This journal is © The Royal Society of Chemistry 2016

Dynamic Acidity in Defective UiO-66

Sanliang Ling[a] and Ben Slater[a]

[a] Department of Chemistry, University College London, 20 Gordon Street, London, WC1H 0AJ, United Kingdom

E-mails: s.ling@ucl.ac.uk; b.slater@ucl.ac.uk

1. Computational details

All periodic calculations were performed using the CP2K code which uses a mixed Gaussian/plane-wave basis set.[1, 2] We employed double-ζ polarization quality Gaussian basis sets and a 600 Ry plane-wave cutoff for the auxiliary grid, in conjunction with the Goedecker−Teter−Hutter pseudopotentials.[3, 4] Structural optimizations, including atomic coordinates and cell parameters, and total energy calculations of the periodic systems, were performed at density functional theory (DFT) level, using both PBE[5] and PBE0[6, 7] functionals, together with Grimme's D3 van der Waals correction[8] with the Axilrod−Teller−Muto three-body terms. Additional calculations (see Table S1) were performed using different DFT functionals on two selected defect configurations (D0 and D1), and we found that the relative energies between D0 and D1 are consistent between different methods. The exact Hartree-Fock exchange calculations, which are part of the hybrid DFT functional PBE0, were performed and significantly accelerated using the auxiliary density matrix method (ADMM),[9] which enables us to consider relatively large systems (with the largest system containing 456 atoms) at hybrid DFT level. A convergence threshold of 1.0×10^{-6} Hartree was used for all self-consistent field (SCF) calculations. The structural optimizations were considered converged if the maximum force on all atoms falls below 0.534 kcal mol^{-1} Å^{-1} (4.5×10^{-4} Hartree Bohr^{-1}). The optimised lattice parameters are 20.918 Å and 20.731 Å at PBE+D3 and PBE0+D3 levels of theory, respectively, both of which are in good agreement with the experimental value of 20.724 Å (at 200 K).[10] Calculations of defective cells were performed with the fixed lattice parameters of

the perfect defect-free cell. The counterpoise method[11] was used to correct for basis set superposition errors (BSSEs) in all binding energy calculations.

Table S1: Relative stabilities (ΔE, in kJ/mol per defect centre) between defect configurations D0 and D1 determined using different DFT functionals.

Method	ΔE
PBE+D3	33.1
PBE0+D3	40.7
BLYP+D3	42.2
HSE06+D3	38.9

Ab initio molecular dynamics (AIMD) simulations within the Born-Oppenheimer approximation were performed in the canonical ensemble, i.e. with constant number of particles, constant volume and constant temperature (NVT), at the PBE+D3 level of theory based on PBE+D3 lattice parameters determined for the perfect defect-free lattice. A time step of 0.5 ps was used for the integration of the equation of motion. The AIMD simulations were performed at four different temperatures, including 100 K, 300 K, 500 K and 700 K, which were controlled by the canonical sampling through velocity rescaling (CSVR) thermostat[12] using a time constant of 50 fs. The simulations at 100 K, 500 K and 700 K were performed for a duration of 10 ps (excluding equilibration), and the simulation at 300 K were performed for a duration to 30 ps (excluding equilibration). All calculations were performed in a conventional unit cell of UiO-66, and the initial structures that were optimized were taken from the experimentally resolved crystal structures UiO-66.[13]

The estimation of the transition barriers for proton transfer was performed based on a linear interpolation (a total of 7 intermediate images) of the atomic coordinates of the initial and final configurations, both of which were fully relaxed, at PBE0+D3 level of theory. Then partial geometry optimisations were performed on intermediate images, in which only hydrogen atoms belong to hydroxide anion and water molecules involved in the proton transfer

were allowed to relax while positions of all other atoms were fixed. Clearly this constrained calculation will yield barriers that overestimated but nevertheless, the transport barriers are already rather small and smaller than many bulk proton activation barriers reported in a wide range of metal-organic frameworks.[14]

2. Analysis of the AIMD trajectories

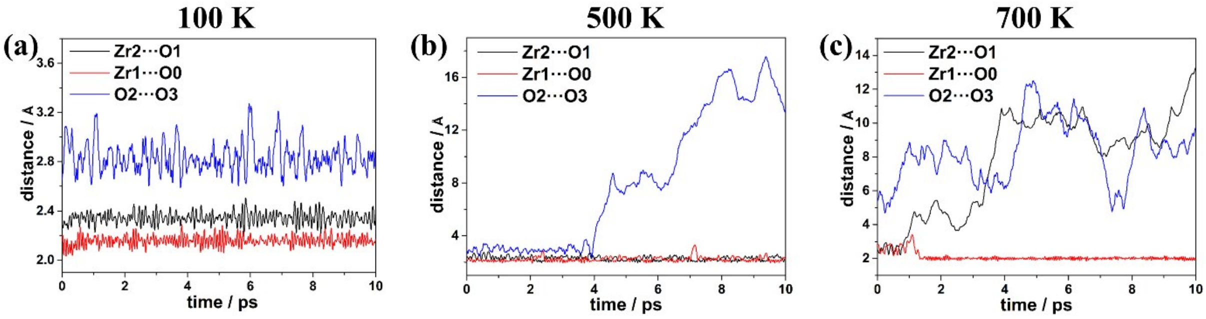

Figure S1: Changes of distances (in Å) during the AIMD simulations starting from D1 at (a) 100 K, (b) 500 K and (c) 700 K.

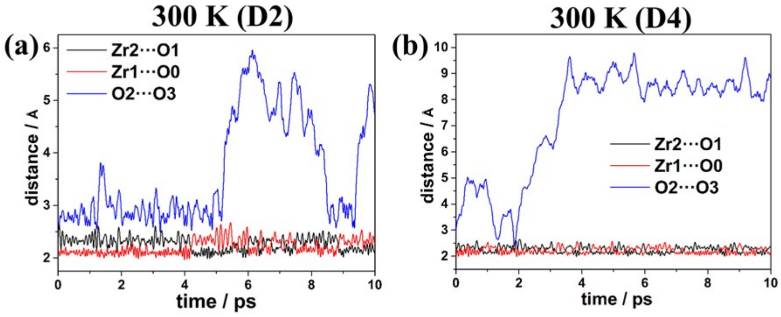

Figure S2: Changes of distances (in Å) during the AIMD simulations starting from (a) D2 and (b) D4[15] at 300 K.

3. Static and AIMD simulation at 300 K starting from propoxide as the charge balancing anion

We started from a geometry similar to the one proposed by Trickett et al.,[10] with a single water molecules coordinated to each of the two Zr atoms at a defect centre, and a

propoxide moiety placed in the vicinity of the μ_3-OH. After geometry relaxation, we found the proton belonging to the μ_3-OH spontaneously transferred to the charge balancing propoxide anion. This is equivalent to what happened in defect configuration D1 where hydroxide rather than propoxide acts as the charge balancing species - see Figure 2b. The O2···O3 distance in the optimised geometry is 2.48 Å, almost the same to that in defect configuration D1 (2.46 Å at PBE+D3 level). Starting from this configuration, we performed AIMD simulation at 300 K for a duration of 10 ps (excluding equilibration), and we show the changes of distances during the AIMD simulation in Figure S3. We found that two proton transfers happened almost simultaneously in less than 1 ps after the start of the AIMD run, i.e. proton in one of the water molecules transferred to the neutral propanol molecule, and another proton in the hydroxide of the neutral propanol molecule transferred back to μ_3-O of UiO-66. This is reflected by the two different Zr···O distances as shown in Figure S3, with the shorter distance corresponding to Zr-OH and the longer distance corresponding to Zr···H_2O. The static and AIMD simulation at 300 K indicate that the charge balancing anion in the case of UiO-66 synthesised from zirconium propoxide is also hydroxide.

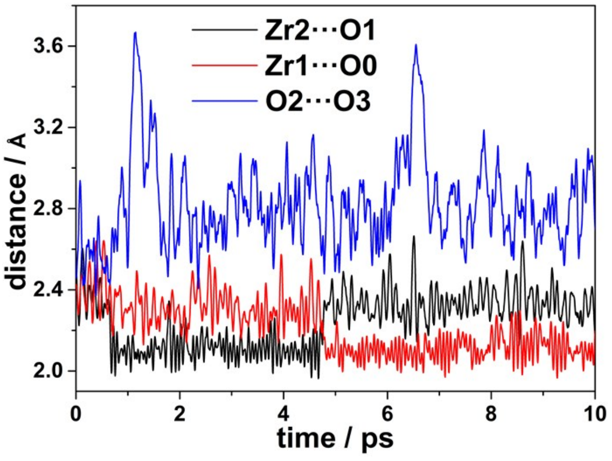

Figure S3: Changes of distances (in Å) during the AIMD simulation at 300 K starting from propoxide as charge balancing anion.

4. Two movies showing the AIMD simulations starting from D1 at 300 K.

5. An input example for AIMD simulation.

References

1 J. VandeVondele, M. Krack, F. Mohamed, M. Parrinello, T. Chassaing and J. Hutter, *Comput. Phys. Commun.*, 2005, **167**, 103-128.
2 J. Hutter, M. Iannuzzi, F. Schiffmann and J. VandeVondele, *WIREs Comput. Mol. Sci.*, 2014, **4**, 15-25.
3 S. Goedecker, M. Teter and J. Hutter, *Phys. Rev. B*, 1996, **54**, 1703-1710.
4 M. Krack, *Theor. Chem. Acc.*, 2005, **114**, 145-152.
5 J. P. Perdew, K. Burke and M. Ernzerhof, *Phys. Rev. Lett.*, 1996, **77**, 3865-3868.
6 C. Adamo and V. Barone, *J. Chem. Phys.*, 1999, **110**, 6158.
7 M. Ernzerhof and G. E. Scuseria, *J. Chem. Phys.*, 1999, **110**, 5029.
8 S. Grimme, J. Antony, S. Ehrlich and H. Krieg, *J. Chem. Phys.*, 2010, **132**, 154104.
9 M. Guidon, J. Hutter and J. VandeVondele, *J. Chem. Theory Comput.*, 2010, **6**, 2348-2364.
10 C. A. Trickett, K. J. Gagnon, S. Lee, F. Gandara, H. B. Burgi and O. M. Yaghi, *Angew. Chem. Int. Ed.*, 2015, **54**, 11162-11167.
11 S. F. Boys and F. Bernardi, *Mol. Phys.*, 1970, **19**, 553-566.
12 G. Bussi, D. Donadio and M. Parrinello, *J. Chem. Phys.*, 2007, **126**, 014101.
13 L. Valenzano, B. Civalleri, S. Chavan, S. Bordiga, M. H. Nilsen, S. Jakobsen, K. P. Lillerud and C. Lamberti, *Chem. Mater.*, 2011, **23**, 1700-1718.
14 S. Tominaka and A. K. Cheetham, *RSC Adv.*, 2014, **4**, 54382-54387.
15 Defect configuration D4 is not shown in Figure 2. It is similar to D0 and D2, but with slightly different hydrogen bonding network.
